# Hypoxia Inducible Factor-2 Alpha and Prolinhydroxylase 2 Polymorphisms in Patients with Acute Respiratory Distress Syndrome (ARDS)

**DOI:** 10.3390/ijms18061266

**Published:** 2017-06-14

**Authors:** Annika Dötsch, Lewin Eisele, Miriam Rabeling, Katharina Rump, Kai Walstein, Alexandra Bick, Linda Cox, Andrea Engler, Hagen S. Bachmann, Karl-Heinz Jöckel, Michael Adamzik, Jürgen Peters, Simon T. Schäfer

**Affiliations:** 1 Klinik für Anästhesiologie und Intensivmedizin, Universitätsklinikum Essen, Hufelandstraße 55, D-45122 Essen, Germany; MiriamRabeling@t-online.de (M.R.); katharina.k.rump@rub.de (K.R.); kai.walstein@gmail.com (K.W.); alexandra.bick@uk-essen.de (A.B.); linda.cox@uk-essen.de (L.C.); andrea.engler@uk-essen.de (A.E.); michael.adamzik@kk-bochum.de (M.A.); juergen.peters@uni-duisburg-essen.de (J.P.); simon.schaefer@uk-essen.de (S.T.S.); 2 Institut für Medizinische Informatik, Biometrie und Epidemiologie, D-45122 Essen, Germany; lewin.eisele@uk-essen.de (L.E.); k-h.Joeckel@uk-essen.de (K.-H.J.); 3 Institut für Pharmakogenetik, Universitätsklinikum Essen and Universität Duisburg-Essen, D-45122 Essen, Germany; hagen.bachmann@uk-essen.de; 4 Klinik für Anästhesiologie, Intensivmedizin und Schmerztherapie; Universitätsklinikum, Knappschaftskrankenhaus Bochum and Ruhruniversität Bochum, In der Schornau 23-25, D-44892 Bochum, Germany; 5 Klinik für Anaesthesiologie, Ludwig-Maximilians-Universität München, Marchioninistrasse 15, D-81377 München, Germany

**Keywords:** acute respiratory distress syndrome, hypoxia inducible factors, prolylhydroxylases, genetic variants, polymorphism

## Abstract

Hypoxia-inducible-factor-2α (HIF-2α) and HIF-2 degrading prolyl-hydroxylases (PHD) are key regulators of adaptive hypoxic responses i.e., in acute respiratory distress syndrome (ARDS). Specifically, functionally active genetic variants of *HIF-2α* (single nucleotide polymorphism (SNP) [ch2:46441523(hg18)]) and *PHD2* (C/T; SNP rs516651 and T/C; SNP rs480902) are associated with improved adaptation to hypoxia i.e., in high-altitude residents. However, little is known about these SNPs’ prevalence in Caucasians and impact on ARDS-outcome. Thus, we tested the hypotheses that in Caucasian ARDS patients SNPs in *HIF-2α* or *PHD2* genes are (1) common, and (2) independent risk factors for 30-day mortality. After ethics-committee approval, 272 ARDS patients were prospectively included, genotyped for *PHD2* (Taqman SNP Genotyping Assay) and *HIF-2α*-polymorphism (restriction digest + agarose-gel visualization), and genotype dependent 30-day mortality was analyzed using Kaplan-Meier-plots and multivariate Cox-regression analyses. Frequencies were 99.62% for homozygous *HIF-2α* CC-carriers (CG: 0.38%; GG: 0%), 2.3% for homozygous *PHD2* SNP rs516651 TT-carriers (CT: 18.9%; CC: 78.8%), and 3.7% for homozygous *PHD2* SNP rs480902 TT-carriers (CT: 43.9%; CC: 52.4%). *PHD2* rs516651 TT-genotype in ARDS was independently associated with a 3.34 times greater mortality risk (OR 3.34, CI 1.09–10.22; *p* = 0.034) within 30-days, whereas the other SNPs had no significant impact (*p* = ns). The homozygous *HIF-2α* GG-genotype was not present in our Caucasian ARDS cohort; however *PHD2* SNPs exist in Caucasians, and *PHD2* rs516651 TT-genotype was associated with an increased 30-day mortality suggesting a relevance for adaptive responses in ARDS.

## 1. Introduction

Acute respiratory distress syndrome (ARDS) is a life-threatening disease characterized by an acute-onset, progressive, hypoxic condition with radiographic bilateral lung infiltration, e.g., due to various pathogens, but not due to hydrostatic pulmonary edema. Thus, acute respiratory distress syndrome (ARDS) results in a highly impaired pulmonary function and hypoxemia [1]. A draft definition proposed three mutually exclusive categories of ARDS based on the degree of hypoxemia: mild (PaO_2_/FIO_2_ ≤ 300 mmHg), moderate (PaO_2_/FIO_2_ ≤ 200 mmHg), and severe (PaO_2_/FIO_2_ ≤ 100 mmHg). ARDS is most often due to direct injury to the lung (e.g., pneumonia and gastric aspiration) or indirect injury (e.g., sepsis and pancreatitis) [2]. However, ARDS can also develop following trauma or inhalation of toxic gases like ozone [3]. Thus, ARDS is characterized by a strong inflammatory response and impaired oxygenation due to a ventilation-perfusion mismatch [4], resulting in pulmonary vasoconstriction caused by the Euler-Liljestrand mechanism. This mechanism, while being helpful in compensating for regional ventilation abnormalities, aggravates hypoxemia in ARDS due to a further increase in pulmonary artery pressure. Of note, in ARDS patients severe pulmonary hypertension can even lead to right heart failure and death. Whereas the Euler-Liljestrand mechanism in ARDS increases pulmonary artery hypertension and hypoxemia, counter regulating mechanisms are activated as well [5]. Key regulators of the hypoxic response are the so-called hypoxia inducible factors (HIF) and HIF degrading prolylhydroxylases (PHD). HIF and PHD are of importance both in hypoxia and inflammation i.e., important during human hypoxemia and profound inflammatory response in ARDS [6,7]. In particular, the HIF-2 pathway is involved in a multitude of biological processes impacting on pulmonary hypertension, erythropoietin synthesis, iron metabolism, bone marrow microenvironment, and tumor progress [8,9,10,11]. Alterations of constitutively activated HIF-2α are associated with an increased risk for neuroendocrine tumors [12,13]. Furthermore, HIF-2α is implicated in the pulmonary regulation of thrombo-spondin-1 and contributes to pulmonary artery hypertension-driven vascular remodeling and vasoconstriction [14]. Thus, differences in HIF-2α activity or HIF degrading prolylhydroxylases might influence the pulmonary artery hypertension counterregulating effects of hypoxemia, and thus alter patients’ outcome.

Recently, functionally active genetic variants were identified both in the *HIF-2α* (C/T; Single Nucleotide Polymorphism SNP C/G [ch2: 46441523(hg18)] and the *PHD2* genes (C/T; SNP rs516651 and T/C; SNP rs480902). These genetic variants were found to be associated with erythrocytosis, pulmonary hypertension, and chronic mountain sickness, respectively [10,15,16,17]. However, it is unknown whether these genetic variants impact on the outcome in ARDS patients.

Accordingly, we tested the hypotheses that (1) SNPs in *HIF-2α* or *PHD2* genes are common in Caucasians, and (2) they are an independent risk factor for 30-day mortality in ARDS.

## 2. Results

### 2.1. Hypoxia Inducible Factor-2α (C/G SNP [ch2: 46441523(hg18)]) Polymorphism

Frequencies were 99.62% for the homozygous CC-genotype, 0.38% heterozygous CG-genotype, and 0% for homozygous GG-genotype. Since none of the ARDS patients in our cohort carried the homozygous GG-genotype no further statistical analyses were performed. In healthy blood donor controls we found the same distribution of genotypes. Thus, the *HIF-2α* GG-genotype was neither present in our ARDS cohort of Caucasian heritage nor in healthy controls.

### 2.2. Prolylhydroxlase 2 (C/T; rs516651) Polymorphism

Allele frequencies were 2.3% for homozygous TT-genotype, 18.9% for heterozygous CT-genotype, and 78.8% for homozygous CC-genotype. Patients’ clinical characteristics did not significantly differ between subcohorts (Table 1), i.e., for serum creatinine concentration or need for dialysis.

**Figure 1:**
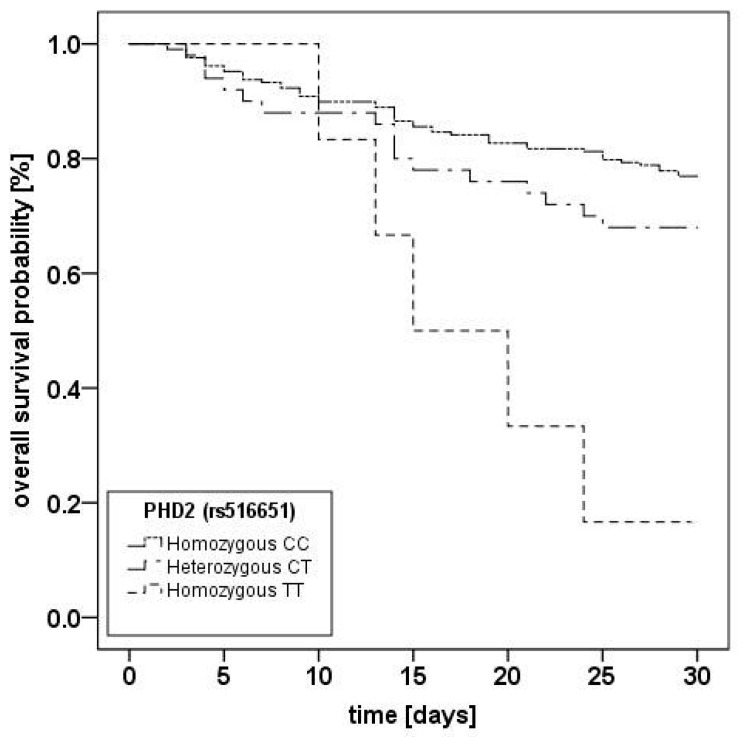
Kaplan–Meier plot of 30-day mortality of acute respiratory distress syndrome (ARDS) patients stratified for *PHD2* (rs516651) genotypes. Kaplan-Meier estimators for the three subgroups for 264 patients. CC = homozygous CC-genotype (*n* = 208); CT = heterozygous CT-genotype (*n* = 50); TT = homozygous TT-genotype (*n* = 6). For 8 patients, genotyping results were unclear and patients were excluded from analysis; log-rank-test: *p* = 0.001.

Kaplan-Meier-analyses (Figure 1) showed a significantly higher mortality for the homozygous TT-genotype (*p* = 0.001).

Most important, multivariate Cox-regression-analyses (Table 2) revealed that the rs516651 TT-genotype is an independent predictor of 30-day mortality from ARDS and carries a 3.34 times higher mortality risk, when including known risk factors for mortality from ARDS like gender, age, need for renal replacement therapy, or SAPS II. Five of 6 (83.3%) ARDS patients with the rs516651 TT-genotype did not survive 30 days.

### 2.3. Prolylhydroxylase 2 (T/C; rs480902) Polymorphism

Allele frequencies were 3.7% for homozygous TT-genotype, 43.9% for heterozygous CT-genotype, and 52.4% for homozygous CC-genotype. Patients’ clinical characteristics did not differ between subcohorts (Table 1). Kaplan-Meier analyses and log-rank test (*p* = 0.199) revealed no impact of the specific genotype on 30-day mortality from ARDS, even in the univariate analysis.

## 3. Discussion

In this study, we show that the functionally active *PHD2* SNP rs516651 [18], located in the key pathway for the hypoxic-inflammatory response, is associated with increased 30-day mortality in ARDS patients. In contrast, the *PHD2* SNP rs480902 is not. Furthermore, the *HIF-2α* SNP [ch2: 46441523(hg18)] GG-genotype was neither present in our ARDS patients of Caucasian heritage nor in healthy Caucasian blood donors.

Hypoxia-inducible factors and HIF degrading PHDs are key regulators of the human response to low ambient oxygen [19] and also provide a link between hypoxia and the inflammatory response [9]. HIF degrading prolylhydroxylase 2 SNPs are known to be associated with erythrocytosis in humans [10] and *PHD2* deficiency evokes erythrocytosis by activating the renal erythropoietin pathway in mice [20]. Furthermore, the lack of *PHD2* leads to defective vascular growth [21]. Moreover, recent studies have shown that genetic variants in *HIF-2α* gene and *PHD2* influence the hypoxic inflammatory response, resulting in altered heart rate, arterial oxygen saturation, incidence of chronic mountain sickness or erythrocytosis [6,22,23].

Our study is the first to analyze the occurrence of these three genetic variants (*HIF-2α* SNP: rs46441523; *PHD2* SNP rs516651 and rs480902) in Caucasians with ARDS and their potential association with death from ARDS.

T-allele carriers of the *PHD2* SNP rs516651 are quite common in Caucasian ARDS patients (TT-genotype: 2.3%; CT-genotype 18.9%) and with homozygous TT-genotype carrying a significantly greater mortality compared to CC-genotypes. As homozygous TT-genotypes were all male patients and older compared to the other genotypes, in a next step we performed a multivariate Cox-regression analysis, including age, gender, need for dialysis as well as other clinical confounders known to carry a higher risk for death from ARDS to our analysis. Thus, even when adjusting for these variables, the TT-genotype of the *PHD2* SNP rs516651 is an independent predictor for 30-day mortality in ARDS. Of note, increased pulmonary artery pressure, often present in ARDS patients and to a varying extent due to hypoxic pulmonary vasoconstriction (the Euler-Liljestrand-mechanism), also is a risk factor for pulmonary edema in high-altitude residents [24]. Of note, genotyping of high-altitude residents adapted to hypoxia, like Tibetans, did not reveal the presence of T-alleles, possibly due to an evolutionary selection bias [15]. Thus, the *PHD2* SNP rs516651 polymorphism may impact on adaptation to hypoxia both in high-altitude-residents and patients with ARDS. However, further studies are needed to confirm our results and to analyze the underlying pathomechanisms. Showing that genetic variants depict a specific phenotype not only in high-altitude residents but also in ARDS patients is crucial before finally judging the relevance of this particular SNP on ARDS pathogenesis.

We also analyzed a further genetic variant in the *PHD2* gene (SNP rs480902) but this genetic variant did not impact on ARDS mortality in our cohort. Wu et al. showed a correlation between single nucleotide polymorphisms in hypoxia-related genes like the *PHD2* (SNPrs480902) and susceptibility to acute high-altitude pulmonary edema (HAPE). They found that the HAPE cases had a significant higher T-allele frequency than the control group [25]. Buroker et al. described phenotypical differences between Han Chinese with acute mountain sickness (AMS) in the rs480902 SNP. They found a significant correlation between (rs480902) SNP (C/T) and heart rate, arterial oxygen saturation of hemoglobin, and the hematocrit in the AMS-group. The CC- and TT-genotypes had a significantly higher heart rate compared to the CT-genotype while patients with the CC-genotype had a significantly greater arterial oxygen saturation of hemoglobin than those with either the CT- or TT-genotypes in the Han AMS study group. AMS Chinese with CT- and TT-genotypes had a significantly higher hematocrit than those with CC-genotypes. It seems that the various genotypes have different mechanisms of compensation in hypoxia. In our study, however, we did not find such differences in the ARDS cohort.

However, mice with *PHD2*-deficiency show increased angiogenesis due to upregulated vascular endothelial growth factor-A (VEGF-A) serum concentrations. Furthermore, erythropoietin, which stimulates angiogenesis and erythropoiesis, was dramatically overexpressed in *PHD2*-deficient mice. Thus, *PHD2* is a major negative regulator for vascular growth. Additionally, *PHD2* knockout in mice inhibits tumor necrosis factor α (TNFα) and intercellular Adhesion Molecule 1 (ICAM-1) expression and decreases both cell apoptosis and macrophage infiltration [21]. Even when *PHD2* alterations impact on the inflammatory response in mice or the hypoxic response in high-altitude residents, analyzed *PHD2* genetic variants did neither impact on serum inflammatory variables like procalcitonin (PCT) or C-reactive Protein (CRP) serum concentrations nor on 30-day mortality from ARDS in our patient cohort. It is known that both hypoxia and inflammation alter the hypoxic-inflammatory response, thus alternative pathways might be induced and could be an effect of those SNPs. To further elucidate this analysis of the genotype dependent *PHD2* protein activity and target gene expression in patients with and without ARDS should be done in a subsequent study.

Third, we analyzed the frequency of the *HIF-2α* gene genetic variant rs46441523. Surprisingly, GG-genotypes did not exist in our Caucasians with ARDS, and only a single (surviving) individual carrying a CG-genotype was observed. Since the frequency distribution of alleles in ARDS patients and blood donors were similar, these data indicate that, at least in our region, this *HIF-2α* polymorphism does not exist in Caucasians either with or without ARDS. It is already know about the *HIF-2α* polymorphism that it changes very fast. Between Tibetan and Han samples one SNP at Endothelial PAS domain-containing protein 1 (EPAS1) shows a 78% frequency difference, representing the fastest allele frequency change observed at any human gene to date [26]. Thus, further studies are warranted to analyze allele frequency distributions in different ethnicities and diseases.

Our study has limitations. First, although we included as many as 272 ARDS patients, our cohort might still be considered small. Second, we included ARDS patients over a long time period, and systemic changes in patient care cannot be entirely ruled out. However, this likely is of minor importance as changes in the standard of care would have influenced all patients similarly irrespective of their genotype, and the intensity in charge was blind to specific patients’ genotypes. In fact, considering this timing, the finding that the homozygous TT-genotype of the *PHD2* SNP rs516651 carried a significantly greater mortality compared to the CC-genotype is even more robust. However, the most important limitation of our study is that we cannot provide mechanistic explanations. Thus, further studies are necessary to analyze genotype-dependent immune cell function, intracellular signaling cascades, and pulmonary vascular tone in ARDS.

The rs480902 SNPs did not meet the Hardy-Weinberg equilibrium, with a *p* Value of 0.01. According to Chen, deviations from the Hardy-Weinberg equilibrium proportions suggest that at least one of the standard underlying assumptions for the test (non-overlapping generations, large population size with random mating, no mutation, no migration, and no selection) may be violated. Thus, the distribution of this genetic variant should be analyzed in a larger cohort.

## 4. Materials and Methods

### 4.1. Patients

The prospective study was reviewed and approved by the Medical Faculty’s ethics committee (No. 06-3078, 1 October 2009, Medical Faculty of the University of Duisburg-Essen, Essen, Germany) and registered in the German clinical trials database (DRKS No.: DRKS00011661). Two hundred seventy two adult patients with ARDS admitted to our intensive care unit (ICU) at the University Hospital Essen, Germany, between 2004 and 2014 were screened for study inclusion. ARDS patients were considered eligible when they fulfilled the definition for ARDS [18]. Patients who refused or withdrew study participation, and all individuals with non-Caucasian ethnicity were excluded.

### 4.2. Samplings

Arterial blood samples were taken for blood tests, microbiology cultures, and later genotyping within the first 24 h after diagnosing ARDS. Furthermore, SAPS II (Simplified Acute Physiology Score II) [27], results of blood cultures, length of hospitalization, and 30-day mortality were assessed [15,28,29]. As the *HIF-2α*-polymorphism was not present in Caucasian ARDS patients, in a second step, we investigated the allele frequencies in a cohort of 100 healthy blood donors (47 females, 53 males, median age: 45 years (Q1; Q3: 30; 59)), following ethics committee approval and informed written consent, to rule out that allele frequencies for the *HIF-2α*-polymorphism vary between ARDS patients and healthy volunteers.

### 4.3. Genotyping

The *PHD2* (T/C) SNP (rs480902) and the *PHD2* (C/T) SNP (rs516651) were detected using Taqman SNP Genotyping Assays (Assay-ID: C___2816291_10; C___2816320_10). Genotyping was performed with a PCR System (StepOnePlus™, Applied Biosystem, Waltham, MA, USA) at standard conditions (60 °C for 30 min followed by 95 °C for 10 s, and for 15 s at 92 °C and with 50 cycles at 60 °C for 1 s). Of 272 patients genotyping the rs516651 SNP we obtained 264 secured genotyping results whereas we were not able to determine genotypes in 8 cases.

The *HIF-2α* (SNP C/G [ch2: 46441523(hg18)]) genotype was determined using restriction digest of a 377 bp PCR amplicon with BsmAI (New England Biolabs, Inc., Ipswich, MA, USA). The SNP was included in its cut site. The digested amplicon resulted in 130 and 247 bp fragments in the presence of the C nucleotide or an uncut 377 bp fragment in the presence of the G nucleotide as Buroker et al. have done it first 2012. The DNA fragment sizes were visualized using a 1.5% Tris-Borat EDTA agarose gel.

### 4.4. Statistical Analyses

Data are presented as medians with interquartile ranges (IQR) unless indicated otherwise. For independent samples the Wilcoxon Kruskal-Wallis signed rank test was used. An a priori alpha error *p* of less than 0.05 was considered statistically significant. In a first step, Hardy-Weinberg-equilibrium was analyzed using the Court Lab calculator 2005–2008 (Court MH; Court-laboratory Hardy-Weinberg calculator; Tufts University; www.tufts.com, Medford, MA, USA for MS Excel) (rs516651; *p* = 0.1611; rs480902; *p* = 0.0127).

Afterwards, we investigated associations between the clinical characteristics of the genetic variants with overall 30-day survival, defined as the interval from time of first diagnosing ARDS until death. Patients alive after the 30-day follow-up were regarded as censored. Kaplan-Meier estimators were used to display the overall 30-day survival data in the respective subcohorts followed by log-rank tests for comparison. Finally, we performed multivariate Cox-regression-analyses to assess the impact of the respective genotypes (*PHD2* rs516651/rs480902), sex, age, Simplified Acute Physiology Score II, Sequential Organ Failure Assessment score, requirement for continuous hemofiltration/dialysis and procalcitonin concentration as predictors for 30-day survival. Statistical analyses were performed using SPSS 21.0 (SPSS Inc., Chicago, USA). For the multivariable Cox-regression-models SAS PHREG Procedure was used (version 9.3, SAS Institute, Cary, NC, USA).

## 5. Conclusions

In conclusion, the genetic SNP rs516651 in the *PHD2* gene is an independent predictor for 30-day mortality in ARDS patients with a Caucasian heritage, whereas the rs480902 SNP is not. Furthermore, we could show that the *HIF-2α* GG-genotype [SNP: ch2: 46441523(hg18)] is not present in Caucasians with or without ARDS, in contrast to Han Chinese. Further studies should be conducted to analyze to what extent the *PHD2* SNP rs516651 alters PHD2 protein expression and activity.

## Figures and Tables

**Table 1:** Clinical characteristics of acute respiratory distress syndrome (ARDS) patients with genetic variants in the *PHD2* gene (SNP rs516651, rs480902) and *HIF-2α*.

Genotype (*n*)	rs516651	rs516651	rs516651	*p* Value	rs480902	rs480902	rs480902	*p* Value	HIF-2α	HIF-2α	*p* Value
	CC (*n* = 208)	CT (*n* = 50)	TT (*n* = 6)	*n* = 264	CC (*n* = 142)	CT (*n* = 119)	TT (*n* = 10)	*n* = 271	CC (*n* = 264)	CG (*n* = 1)	*n* = 265
Gender (women/men; *n*; %)	89/119 (42.8/57.2)	18/32 (36/64)	0/6 (0/100)	0.084 **	54/88 (38/62)	48/71 (40/60)	8/2 (80/20)	0.033 **	107 /157 (59.5/40.5)	1/0 (100/0)	0.410
Age (years; median; Q1; Q3) ^#^	44 (33–55)	45 (35–57)	65.5 (56–71)	0.011 *	45 (34–57)	43 (32–54)	43 (34–50)	0.290 *	44 (33–56)	23	0.178
Height (cm; median; Q1; Q3) ^#^	175 (165–180)	178 (168–185)	178 (171–185)	0.160 *	175 (167–180)	175 (165–183)	170 (163–175)	0.190 *	175 (165–180)	192	0.098
Body weight (kg; median; Q1; Q3) ^#^	85 (70–90)	80 (70–94)	80 (70–90)	0.644 *	85 (70–90)	85 (70–93)	78 (65–80)	0.322 *	85 (70–90)	86	0.782
Body mass index (kg·m^−2^; median; Q1; Q3) ^#^	26.7 (23.4–30.5)	26.3 (23.9–29.4)	24.2 (23.5–26.3)	0.389 *	26.1 (23.4–29.4)	27.3 (23.9–31.2)	26.4 (21.9–28.5)	0.436 *	26.3 (23.9–30.1)	23.3	0.322
Mean arterial blood pressure (mmHg; median; Q1; Q3) ^#^	80 (72–87)	83 (75–88)	86 (68–98)	0.472 *	80 (72–87)	81 (73–87)	95 (87–121)	0.046 *	110 (90–125)	-	-
Mean systolic arterial blood pressure (mmHg; median; Q1; Q3) ^#^	120 (110–130)	120 (115–130)	136 (128–150)	0.126 *	120 (110–129)	120 (110–130)	121 (115–145)	0.330 *	80 (72–87)	-	-
Heart rate (min^−1^; median. Q1; Q3) ^#^	107 (90–123)	113 (99–130)	106 (77–148)	0.648 *	110 (95–120)	110 (90–132)	100 (82–114)	0.569 *	120 (110–130)	110	0.334
Mean pulmonary arterial pressure (mmHg; median; Q1; Q3) ^#^	35.5 (31–40)	33 (28–39)	38.5 (34–42)	0.261 *	35 (30–41)	35 (29–40)	38 (35–45)	0.416 *	35.5 (30–41)	29	0.321
Lower airway pressure (median; Q1; Q3) ^#^	18 (15–20)	18 (14–20)	16 (10–18.5)	0.468 *	18 (15–20)	18 (15–20)	15 (10–20)	0.359 *	18 (15–20)	22	0.126
Horovitz ratio (median; Q1; Q3) ^#^	108 (73–195)	95 (72–172)	146 (98–193)	0.614 *	110 (77–201)	106 (68–178)	96 (77–139)	0.517 *	107 (73–187)	115	0.642
Creatinin serum concentration (mg·dL^−1^; median; Q1; Q3) ^#^	1.42 (1.00–2.43)	1.27 (0.96–1.89)	1.34 (1.16–1.48)	0.413 *	1.48 (1.01–2.17)	1.33 (1.00–2.75)	1.00 (0.82–1.76)	0.274 *	1.4 (1–2.4)	1.11	0.225
Dialysis (yes/no; %)	115/76 (60.2/39.8)	21/24 (53.3/46.7)	5/1 (83.3/16.7)	0.114 **	81/46 (63.8/36.2)	61/51 (54.5/45.5)	5/5 (50/50)	0.288 **	145/98 (59.7/40.3)	0/1 (0/100)	0.759
Infectious variables											
White blood cell count (10^9^·L^−1^; median; Q1; Q3) ^#^	14.2 (8.9–21.9)	13.4 (9.4–22.2)	9.8 (8.8–15.6)	0.517 *	14.5 (9.2–22.9)	13.4 (8.8–21.1)	12.8 (8.6–22.3)	0.785 *	13.8 (8.8–21.8)	16.4	0.357
Procalcitonin serum concentration (µg·L^−1^; median; Q1; Q3) ^#^	4.48 (0.69–28.70)	2.56 (0.85–11.9)	0.71 (0.29–30.13)	0.801 *	2.21 (0.60–16.67)	5.38 (0.93–37.38)	5.34 (1.01–68.55)	0.139 *	3.78 (0.69–26.4)	-	-
C-reactive protein concentration (g·L^−1^; median; Q1; Q3) ^#^	19.9 (13.3–27.5)	17.4 (7.1–28.3)	18.4 (12.8–37.3)	0.435 *	18.4 (11.50–24.8)	20.7 (12.8–32.7)	19.0 (8.3–24.1)	0.286 *	19.6 (11.8–27.8)	28.2	0.967
Disease severity											
SAPS II ^##^ (mean ± SD) ^+^	42.5 (30–58)	39.5 (30–60)	41.5 (32–71)	0.836 *	43 (31–61)	42 (30–60)	27 (19–42)	0.099 *	43 (31–60)	42	0.482
SOFA ^++^ (mean ± SD) ^+^	14 (11–20)	13 (10–16)	13.5 (10–20)	0.368 *	15 (12–22)	13 (10–18)	14 (11–22)	0.095 *	14 (11–20)	12	0.433
Hospital stay (d; mean ± SD) ^+^	22 (14–38)	22 (14–34)	18 (13–24)	0.742 *	24 (13–39)	21 (14–33)	18 (14–26)	0.596 *	22 (14–37)	14	0.548
30-day mortality (%)	48 (23)	16 (32)	5 (83.3)	0.002 **	39 (27.5)	32 (26.9)	0 (0)	0.158 **	70 (26.5)	0 (0)	-

Biometric data, infectious variables and disease severity of 264 ARDS patients. Data were documented at the time of first diagnosing ARDS. * Numbers (*n*); *p* Value based on Kruskal-Wallis-Test; ^+^ mean ± standard deviation; ^#^ median with 25% and 75% quartiles (median; Q1; Q3); ** Numbers; *p* Value based on Pearson-Chi-quadrat tests; ^##^ Simplified Acute Physiology Score II; ^++^ Sequential Organ Failure Assessment score.

**Table 2:** Cox-regression analyses of 264 ARDS patients with genetic variants in the Prolylhydroxylase 2 gene (rs516651).

Covariables	HR (95% CI)	*p* Value
rs516651, CT vs. CC	1.71 (0.89–3.29)	0.11
rs516651, TT vs. CC	3.34 (1.09–10.22)	0.034
Age, per year	1.01 (0.99–1.036)	0.40
Gender, male vs. female	0.92 (0.49–1.74)	0.80
Dialysis, yes vs. no	0.94 (0.44–2.01)	0.87
SAPS II, per point	1.03 (1.01–1.04)	0.0032
SOFA	1.01 (0.98–1.04)	0.43
PCT	1.002 (1.00–1.003)	0.038

Number of patients with missing data for the different covariables were: Dialysis (*n* = 22), Simplified Acute Physiology Score II (SAPS II) (*n* = 12), Sequential Organ Failure Assessment score (SOFA) (*n* = 12), procalcitonin (PCT) (*n* = 67). The Table displays hazard ratio (HR) point estimates, 95% confidence intervals (95% CI), and *p* Values derived from Cox–regression- analyses.
